# Intradermal Injection of Oxytocin Aggravates Chloroquine-Induced Itch Responses *via* Activating the Vasopressin-1a Receptor/Nitric Oxide Pathway in Mice

**DOI:** 10.3389/fphar.2019.01380

**Published:** 2019-11-15

**Authors:** Rulong Li, Hua Sun, Haotian Zheng, Zhihua Zong, Shengnan Li, Tingting Meng, Jing Li, Yunfang Liu, Chao Wang, Jingxin Li

**Affiliations:** ^1^Department of Physiology, School of Basic Medical Sciences, Shandong University Cheeloo Medical College, Jinan, China; ^2^Department of the Sixth Internal Medicine, Shandong Cancer Hospital and Institute, Shandong First Medical University and Shandong Academy of Medical Sciences, Jinan, China; ^3^Department of Pathology, Central Hospital of Zibo, Zibo, China; ^4^Center for Strategic Studies, Chinese Academy of Engineering, Beijing, China; ^5^Department of Rehabilitation Medicine, Shandong Provincial Hospital affiliated to Shandong University, Jinan, China

**Keywords:** oxytocin, itch, vasopressin-1a receptor, chloroquine, nitric oxide

## Abstract

Oxytocin (OT), a hormone synthesized within the paraventricular nucleus and supraoptic nucleus of the hypothalamus, when given intracerebroventricularly, induces strong scratching behaviors. However, it is not clear whether intradermal injection (ID) of OT elicits itch sensation. Herein, we found that OT (0.02 mg/ml) did not elicit an itch-scratching response in mice but aggravated chloroquine (CQ, 3 mmol/L)-elicited scratching behavior. Similar to OT, arginine vasopressin (AVP, 0.02 mg/ml), which is structurally related to OT, also enhanced CQ-induced scratching behavior but did not directly induce scratching behavior in mice. Mechanistically, OT-mediated enhancement of CQ-induced scratching behavior was significantly suppressed by conivaptan (0.05 mg/ml), a vasopressin-1a receptor (V1AR) antagonist and 1,400 W (3 mg/kg), inhibitor of inducible nitric oxide synthase (iNOS), but not OT receptor (OTR) antagonist L-368,899 (0.05 mg/ml). Notably, conivaptan also directly decreased CQ-induced scratching. In conclusion, OT plays a role in CQ-induced scratching behavior *via* V1AR binding events. V1AR antagonists could be used as possible treatments for CQ-induced itch.

## Introduction

Oxytocin (OT) is a peptide hormone produced by the paraventricular nucleus and supraoptic nucleus of the hypothalamus. It is transported to the posterior pituitary and secreted into the bloodstream, exerting various hormonal effects. OT can also be released into the central nervous system (CNS), acting as a modulator of neuronal transmission (Leng and Ludwig, 2008; Raggenbass, 2008). Recently, because of social and sexual behavior regulatory functions in mammals, including humans, OT and arginine vasopressin (AVP) have received increased attention and interest (Baumgartner et al., 2008; Donaldson and Young, 2008; Walum et al., 2008; Young et al., 2008). OT and AVP are closely related and can both be recognized by two receptors, oxytocin receptor (OTR), and vasopressin-1a receptor (V1AR) (Swanson and Kuypers, 1980; Sofroniew, 1983; Chini and Manning, 2007). Concurrently, an increased number of studies have approved the analgesic effects of OT for humans and rodent species from a variety of pain tests (Honda and Takano, 2009; Koshimizu and Tsujimoto, 2009). OT has shown pain suppression effects when it is administered in the brain, spinal cord, or systemically (Lundeberg et al., 1994; Ge et al., 2002; Wang et al., 2003; Yu et al., 2003; Gao and Yu, 2004; Miranda-Cardenas et al., 2006; Reeta et al., 2006; Condes-Lara et al., 2007). In addition, OT injection in the CNS induces strong scratching and grooming behaviors in mice and rats (Meisenberg and Simmons, 1982; Van Wimersma Greidanus et al., 1990; Liu et al., 2009). Similar to OT, central administration of AVP robustly stimulated stereotypical scratching and autogrooming in mice (Bielsky et al., 2004). However, it is not clear whether intradermal injection (ID) of OT or AVP elicits scratching behavior. Chloroquine (CQ), the antimalarial drug, also induces itch in humans and scratching in mice (Green et al., 2006) *via* a histamine-independent pathway linked to activation of Mas-related G protein-coupled receptors (Mrgprs) (Liu et al., 2009). CQ has been used in animal studies and in humans as a tool to study itch mechanisms. Nitric oxide (NO) has been proven to participate in CQ-induced scratching (Foroutan et al., 2015). Interestingly, OT also stimulated NO production in rat dorsal root ganglia (DRG) (Gong et al., 2015). Here we reported, although ID of OT or AVP did not trigger grooming or scratching behavior in mice, it did aggravate CQ-induced scratching behavior in mice. Mechanistically, we revealed that V1AR and NO are involved in OT-mediated enhancement of CQ-induced scratching behavior. Importantly, the V1AR antagonist conivaptan remarkably suppressed CQ-induced scratching, suggesting V1AR antagonists could be used as new therapeutic perspectives for the treatment of CQ-induced itch in human beings.

### Experimental Procedures

#### Animals

Male C57BL/6 mice, aged 6–8 weeks and weighing 22–25 g were used. Mice were housed in facilities with proper temperature (23–25°C) and lighting from 08:00 a.m. to 08:00 p.m. Mice had free access to food and water. Operational guidelines in facilities, routine husbandry, handling, and experimental procedures were approved by the committee for animal ethics and experiments at Shandong University, Jinan, China.

#### Drugs

CQ was purchased from Sigma (St. Louis, MO, USA). OT, histamine, AVP, conivaptan, and L-368,899 were bought from MCE (Monmouth Junction, New Jersey, USA).The inducible nitric oxide synthase (iNOS) blocker 1,400 W was obtained from APExBIO (Houston, TX, USA). All the drugs were dissolved in normal saline (NS) except conivaptan and L-368,899 which were mixed with 10% dimethyl sulfoxide (DMSO) as stock solutions and then diluted in NS to a final concentration of 0.05 mg/ml (as a result, dissolved in 0.1% DMSO) immediately before use, respectively.

#### Behavioral Experiments

Mice were placed and habituated in a carton box (45 x 20 x 35 cm) at 23°C ± 1°C for 1 h for three consecutive days before the behavioral experiments. Hair was removed from the rostral back of each mouse by depilatory cream on the day of habituation. On the fourth day, the mice were removed from the box, and 20 µl CQ or histamine was delivered to each mouse intradermally once in the shaved area. To observe the effects of drugs on CQ- or histamine-induced response, each drug was injected 5 min before CQ or histamine treatment. After the injection, the mice were placed back in the same box, and their behavior was recorded for 30 min by a video camera in unmanned conditions to avoid interference. The video was replayed and used for quantification of scratching bouts directed at the site of injection. One scratching bout was counted when the mouse lifted the hind paw to the injection area and returned the same hind paw to the floor or to the mouth.

#### Measurement of Oxytocin Levels in Skin Tissue

Mice were sacrificed by cervical dislocation. The rostral skin (site of injection) was removed from each mouse 15 min after ID injection of CQ and saline. Next, we evaluated the changes in OT concentration after injection of CQ (300 µg, ID). OT was measured by enzyme-linked immunosorbent assay (ELISA) in the homogenized supernatant samples.

#### Statistical Analysis

All statistical analyses were carried out using SPSS (version 19.0; SPSS, Chicago, IL, USA). The data are presented as the means ± SEM; n is the number of mice examined. For the comparison of two groups, independent-samples *t*-test was used. *P*-values <0.05 were considered statistically significant.

## Results

### Intradermal Injection of Oxytocin Did Not Induce Scratching Behavior in Mice

**Figure 1:**
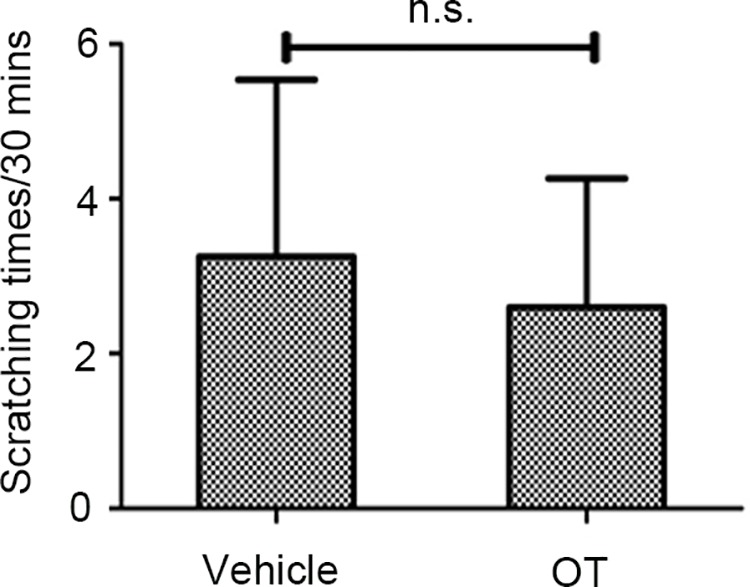
The effect of oxytocin (OT) (0.4 µg/site, intradermal injection) on scratching behavior. An independent-samples *t*-test presents the effect of intradermal OT (*p* > 0.05). Intradermal OT (0.4 µg/site, n = 10) does not induce significant scratching behavior. Vehicle, normal saline. n.s., no significance.

In the behavioral experiment, ID of OT (0.4 µg/site) alone did not generate significant enhancement in the scratching behavior compared with NS (P > 0.05).These results reveal that OT does not directly contribute to scratching behavior in mice (**Figure 1**, (**Supplemental Video 1**, OT only).

### Oxytocin Markedly Augments Chloroquine-Induced Scratching Levels, But Not Histamine-Induced Scratching Behavior

**Figure 2:**
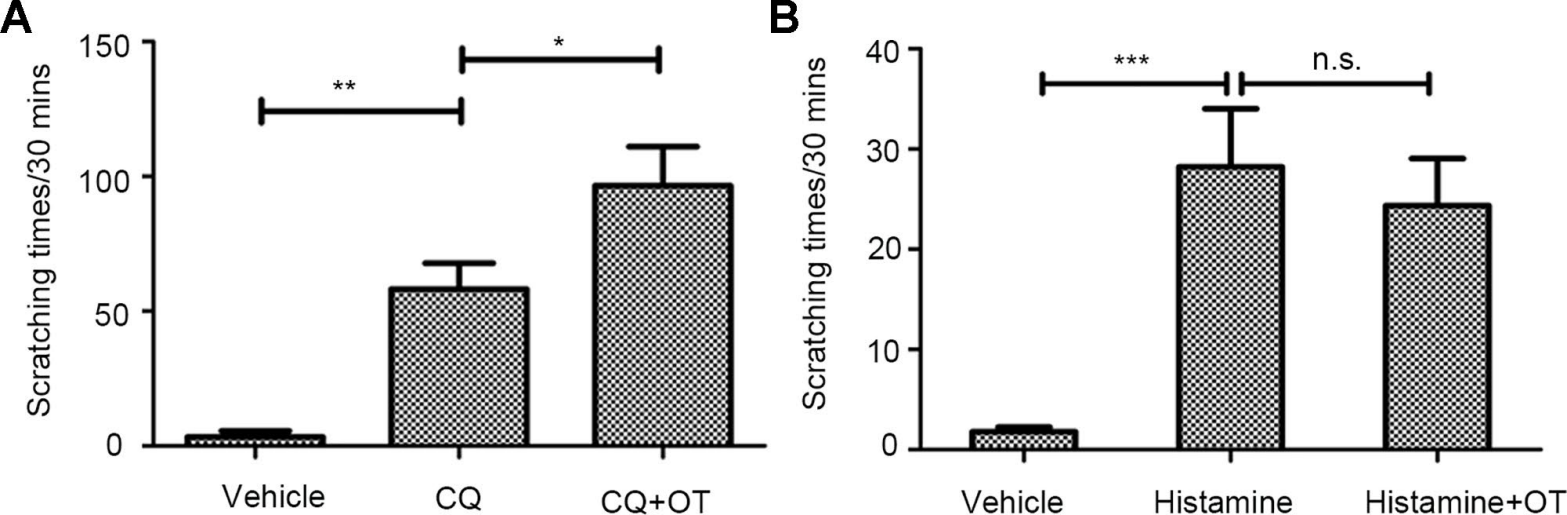
The effects of oxytocin (OT) on chloroquine (CQ)- or histamine-evoked scratching behavior. An independent-samples *t*-test presents the effect of CQ (300 µg/site, n = 10, **:p < 0.01), OT × CQ interaction (0.4 µg/site, n = 6, *:*p* < 0.05), and OT ×histamine interaction (0.1%, 20 µl/site, n = 5, *P* > 0.05). CQ evoked a significant scratching behavior. Notably, OT markedly increases CQ-induced scratching levels **(A)**, but not histamine-evoked scratching levels **(B)**. Vehicle, normal saline. ***: *p* < 0.001. n.s., no significance.

In the follow-up experiment, compared with scratching times of mice injected with CQ (300 µg/site) alone (**Supplemental Video 2**, NS+CQ), mice with OT pretreatment showed significantly higher average scratching times (*P* < 0.05) (**Figure 2A**, **Supplemental Video 3**, CQ+OT). That OT significantly potentiates the CQ (300 µg/site)-induced scratching behavior indicates that OT may play a role in the pruritic pathway of CQ.

Histamine can also induces itch. Therefore, we were curious whether OT would increase the pruritic behavior stimulated by histamine. As a result, OT (0.4 µg/site) administration before histamine (0.1%, 20 µl/site) delivery did not affect the scratching behavior significantly (p > 0.05) (**Figure 2B**).

### The Potentiation Effect of Oxytocin on Chloroquine-Induced Pruritic Behavior Is Mediated by Vasopressin-1a Receptor Rather Than Oxytocin Receptor

**Figure 3:**
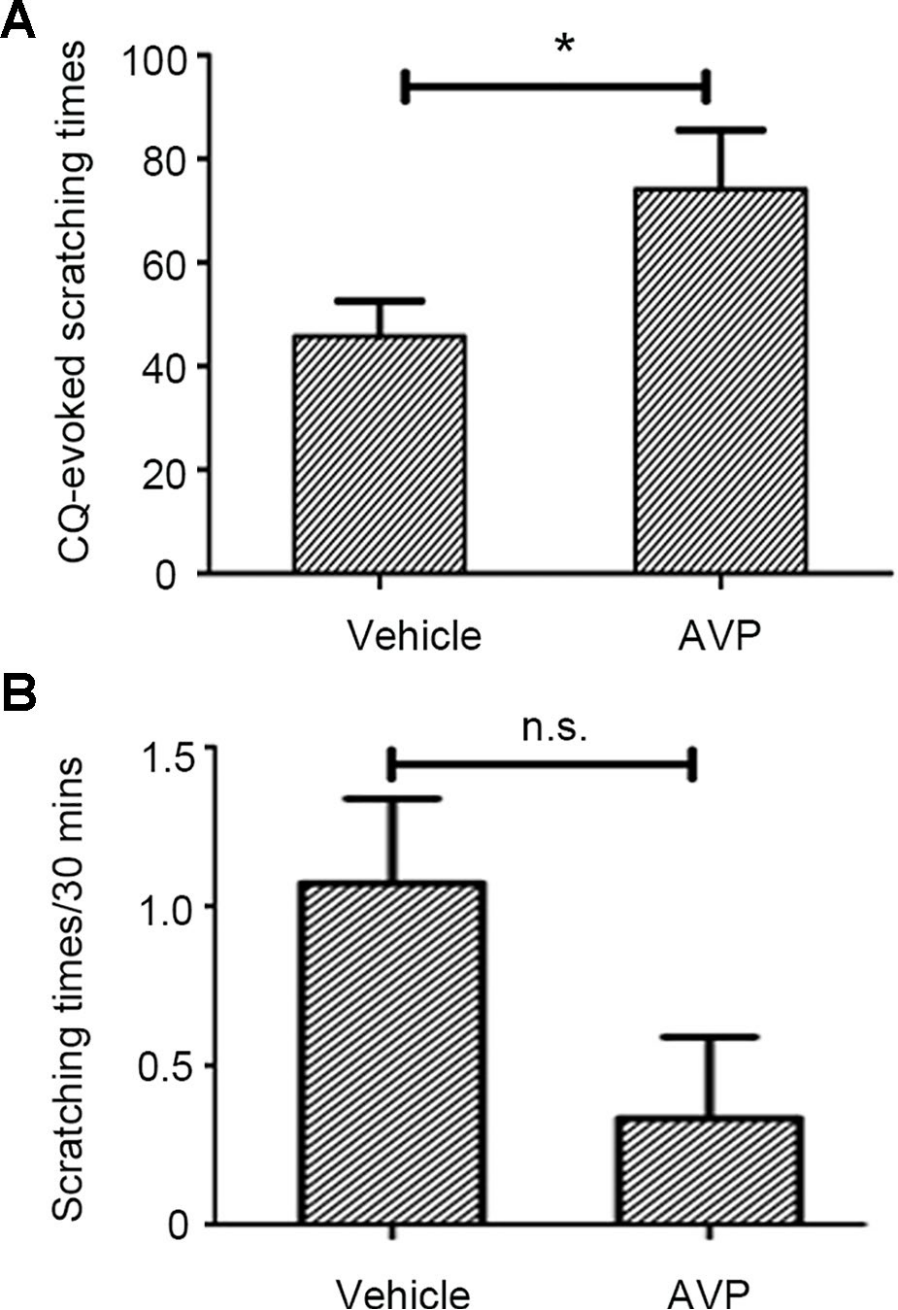
The effect of arginine vasopressin (AVP) on chloroquine (CQ)-evoked Scratching Behavior. Similar to OT, intradermal injection of AVP (0.4 µg/site, n = 7) enhanced CQ-induced scratching behavior **(A)** but AVP only did not induce scratching behavior in mice **(B)**. Vehicle, normal saline. n.s., no significance, *: *p* < 0.05.

ID of AVP (0.4 µg/site) significantly enhanced CQ-induced scratching behavior (**Figure 3A**, **Supplemental Video 4**, CQ+AVP) while it did not induce scratching behavior when administered alone in mice (**Figure 3B**, **Supplemental Video 5**, AVP only). AVP potentiated CQ-induced scratching behavior similar to OT, which implies that a common receptor of the two peptides could be involved in this facilitation.

**Figure 4:**
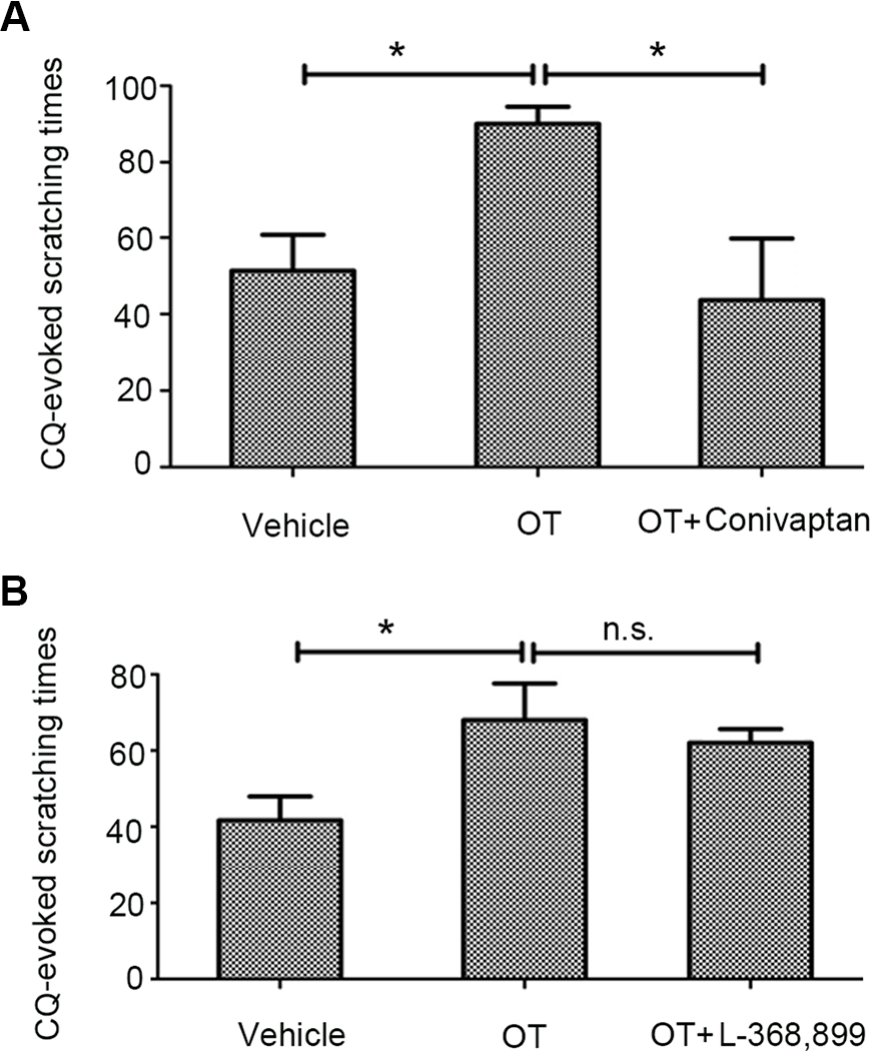
The effects of the oxytocin receptor (OTR) antagonist or vasopressin-1a receptor (V1AR) antagonist on OT-induced enhancement. In the presence of conivaptan (1 µg/site, n = 5), a selective V1AR antagonist, markedly blocked OT-induced augment of scratching behavior induced by CQ **(A)**. However, pretreatment with the OTR antagonist L-368,899 (1 µg/site, n = 4) did not show any suppression effect **(B)**. Vehicle, 0.1% dimethyl sulfoxide. n.s., no significance, *: *p* < 0.05.

To further address the hypothesis of the common receptor, we blocked the effect of peripheral endogenous OT and observed its effect on CQ-induced scratching behavior through behavioral experiments. Notably, the OT-mediated enhancement of CQ-induced scratching behavior was significantly suppressed in mice pretreated with the V1AR antagonist conivaptan (1 µg/site) (**Figure 4A**, **Supplemental Video 6**, conivaptan+OT+CQ). However, mice pretreated with the OTR antagonist L-368,899 (1 µg/site) did not show any suppression effect (**Figure 4B**, **Supplemental Video 7**, CQ+OT+L-368899).

### The Inducible Nitric Oxide Synthase Blocker 1,400 W Greatly Reduces Chloroquine-Induced Scratching Behavior

**Figure 5:**
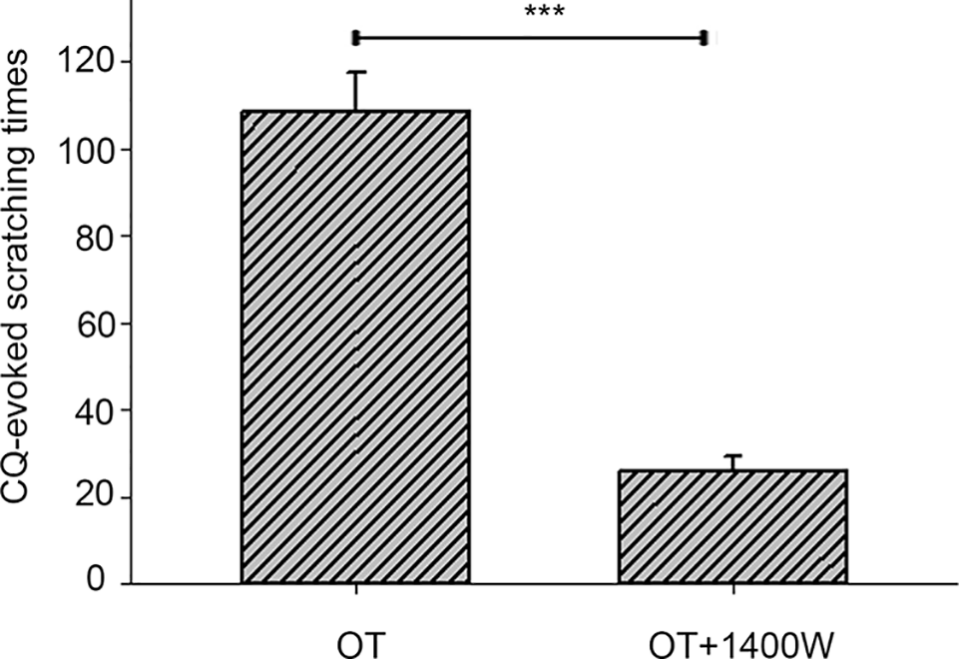
The effect of inducible nitric oxide synthase (iNOS) blocker 1,400 W on the enhancement of chloroquine (CQ)-induced itch responses by oxytocin (OT). The OT-evoked enhancement on CQ-induced scratching behavior was largely alleviated by pretreatment with the iNOS blocker 1,400 W (3 mg/kg, n = 5). ***: *p* < 0.001.

The OT-mediated enhancement of CQ-induced scratching behavior was largely alleviated by pretreatment with the iNOS blocker 1,400 W (3 mg/kg) (**Figure 5**, (**Supplemental Video 8**, CQ+OT+1,400 W).

### The Vasopressin-1a Receptor Antagonist Conivaptan Alleviates Chloroquine-Induced Scratching Behavior

**Figure 6:**
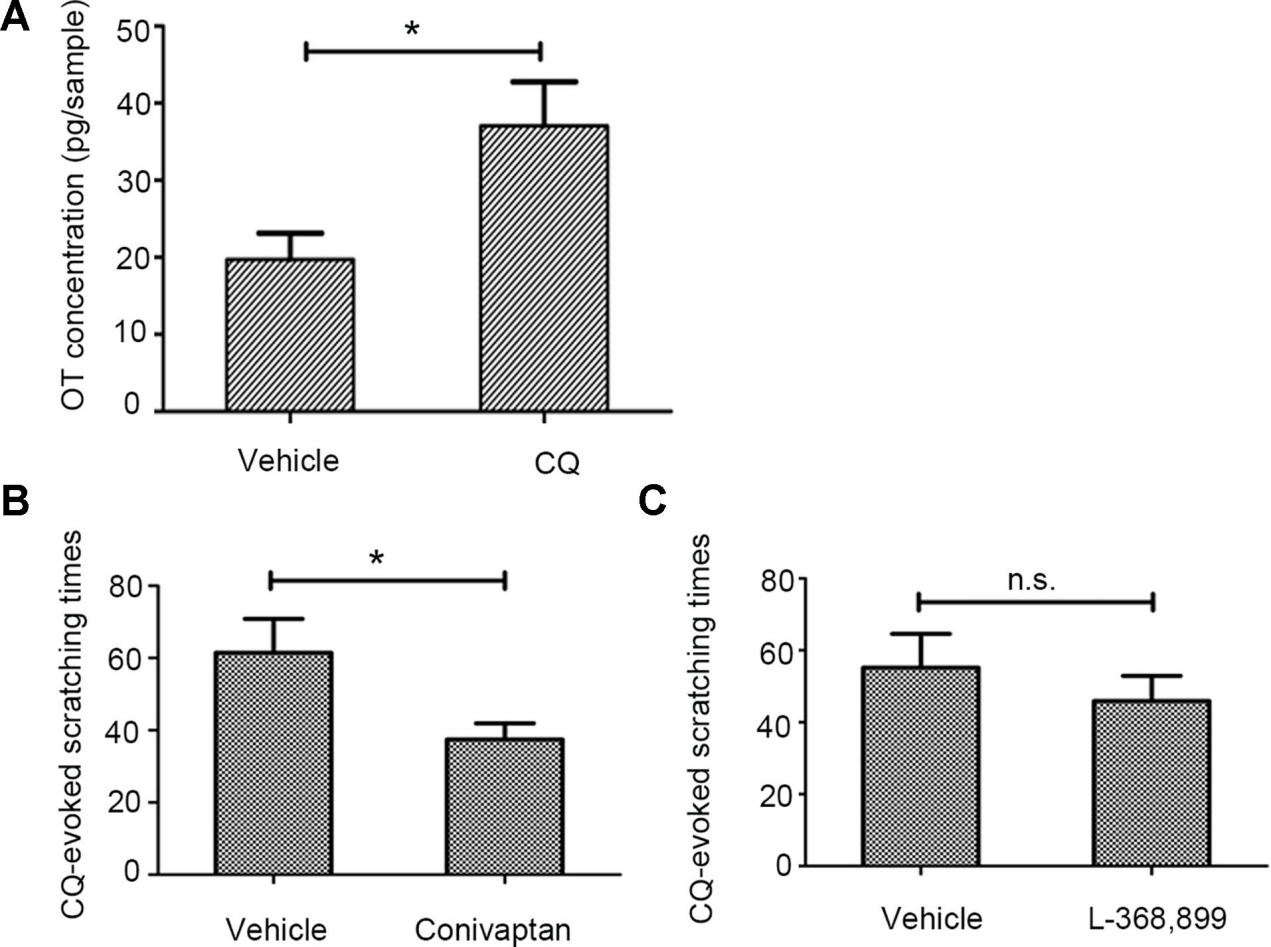
The effect of vasopressin-1a receptor (V1AR) antagonist conivaptan on chloroquine (CQ)-induced scratching behavior. CQ increased the concentration of OT in the injection site of skin (300 µg/site, n = 7, vehicle: normal saline, **A)**. The V1AR antagonist conivaptan (1 µg/site, n = 4, **B)**, but not the OTR antagonist L-368,899 (1 µg/site, n = 5, **C)** significantly alleviated CQ-induced pruritic behavior. Vehicle: 0.1% dimethyl sulfoxide. n.s., no significance, *: *p* < 0.05.

Given the evidence that OT was associated with CQ-induced itch, we then asked whether CQ administration could increase only OT level in the skin. The experimental results showed that ID of CQ increased the concentration of OT but not AVP (**Figure 6A**, **Supplemental Figure 1**). The V1AR antagonist conivaptan (**Supplemental Video 9**, CQ+conivaptan), but not the OTR antagonist L-368,899 (**Supplemental Video 10**, L-368899+CQ) significantly decreased pruritic behavior after 20 µg/site injection of CQ (**Figures 6B, C**).

## Discussion

In our study, we found that OT potentiated CQ-induced scratching behavior *via* binding to the V1AR receptor, and endogenous OT mediated CQ-induced itching.

Previous studies have shown that intracerebroventricular injection of OT elicits scratching behavior in mice (Meisenberg and Simmons, 1982), we asked whether ID of OT would induce scratching behavior. Based on our experimental results, ID of OT does not evoke scratching behavior in mice. CQ is a medication that has been widely used for malaria, certain viral infection (Savarino et al., 2003), systemic lupus erythematosus (Borba et al., 2004), and rheumatoid arthritis treatment (Romanelli et al., 2004). Through the activation of Mas-related G protein-coupled receptors (Liu et al., 2009), CQ induces itch in both mice and humans *via* a histamine-independent pathway (Sowunmi et al., 1989; Green et al., 2006).In this study, we found that ID injection of OT aggravated the CQ-induced itch. Although previous studies have proven that CNS injection of OT into the third ventricle (Schorscher-Petcu et al., 2010) or spinal cord (Thurston et al., 1992) can evoke scratching behavior in mice, our findings first revealed that ID injection of OT could modulate CQ-induced itch.

In terms of the signaling pathway, human MRGPRX1 and the homologous mouse receptor MrgprA3 are activated by CQ (Liu et al., 2009). MrgprA3 couples to Gβγ and modulates TRPA1, which is a Ca^2+^-permeable nonselective cation channel, resulting in itch (Liu et al., 2009; Bautista et al., 2014). In addition, phospholipase Cβ (PLCβ) is also required for CQ-induced itch (Ru et al., 2017). OT has central and peripheral effects that are mediated by the binding event between OT and a single isoform of the OTR (Gimpl and Fahrenholz, 2001). The binding between OT and OTR can also activate the PLCβ signaling transduction pathway (Arnaudeau et al., 1994; Ku et al., 1995; Phaneuf et al., 1995), triggering the release of intracellular Ca2^+^ ([Ca^2+^]_i_) (Chen et al., 2015). Interestingly, our studies found that AVP also enhanced CQ-induced itch and V1AR antagonist significantly alleviated the OT-mediated response. OT and AVP are two nonapeptides that are closely related, and both are produced in the paraventricular and supraoptic nuclei of the hypothalamus (Swanson and Kuypers, 1980; Sofroniew, 1983). Notably, both OT/AVP and OTR/V1AR display high degrees of sequence homology. Both peptides can activate each receptor (Chini and Manning, 2007). OT binds to not only OTR, but also various AVP receptors such as V1A, V1B, and V2 receptors (Vrachnis et al., 2011). AVP predominately exhibits its effect through V1AR, which is coupled to the PLCβ signaling pathway (Schoneberg et al., 1998; Thibonnier et al., 1998; Birnbaumer, 2000). Activation of V1AR stimulates PLCβ and results in production of IP3 and diacylglycerol, leading to an increase in [Ca^2+^]_i_ (Briley et al., 1994). Those mentioned above indicate that OT and CQ share a common signaling pathway. It is possible that OT enhances [Ca^2+^]_i_ levels by activating V1AR and promotes CQ-induced itch. This promotion occurs in addition to the CQ-evoked increase in [Ca^2+^]_i_. Notably, OT did not affect histamine-induced itch. It is known that histamine induces itch by activating H1R and H4R (Shim and Oh, 2008). Furthermore, Shim and colleagues showed that histamine evokes scratching behavior by activating PLA_2_, lipoxygenase, and TRPV1 signaling pathway (Shim et al., 2007). We speculate that OT enhances CQ-but not histamine-induced itch may be associated with their sharing a common signaling pathway with other.

In addition, NO over-production by activating NOS enzymes, especially iNOS contribute to pruritus. Intraperitoneal (IP) treatment of mice with non-selective NOS inhibitor N-nitro-l-arginine methyl ester (l-NAME) and iNOS inhibitor aminoguanidine (AG) markedly inhibited scratching behavior (Ostadhadi et al., 2016). NO might play an important role in CQ-induced scratching. Since NO synthesis is stimulated by CQ in certain cell types (Ghigo et al., 1998; Chen et al., 2005) and CQ-induced scratching is mediated by the NO/cyclic guanosine monophosphate pathway in mice (Foroutan et al., 2015), it is plausible that CQ induces NO production thereby triggering itch.

Our previous studies showed that OT increases NO production in rat DRG (Gong et al., 2015) and enteric neurons (Li et al., 2015). Therefore, the enhancement of the CQ-induced itch by OT was expected to occur through increased NO production, which is also promoted by CQ. As expected, the iNOS blocker largely reduced the OT-mediated enhancement in CQ-induced itch. Notably, pretreatment with the V1AR antagonist significantly alleviated CQ-induced itch, which suggested endogenous OT or AVP may involve in CQ-induced itch. Previous studies have demonstrated that small- and medium-diameter neurons in mouse DRG abundantly express V1AR but not OTR (Schorscher-Petcu et al., 2010), providing a reasonable explanation for our results.

**Figure 7:**
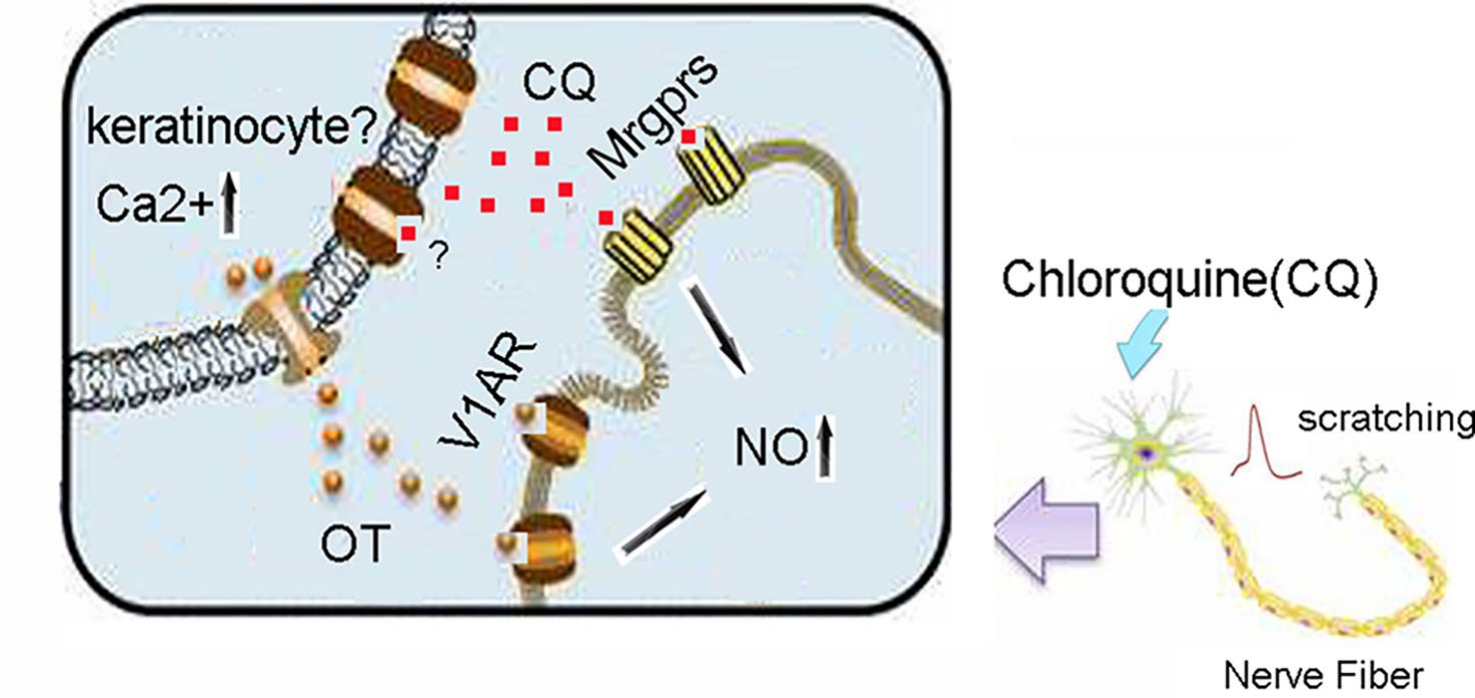
A working model of CQ, OT, V1AR, and Mas-related G protein-coupled receptors (Mrgprs) in mice skin. CQ activates Mrgprs and excited the afferent nerve fibers in the skin, then induces scratching behavior. In addition, CQ also stimulates the release of OT from keratinocytes (a source of local OT), which potentiates CQ-induced itch *via* triggering NO release through activation of V1AR signaling pathway. CQ, chloroquine; OT, oxytocin; V1AR, vasopressin-1a receptor; NO, nitric oxide.

It is well known that peripheral organs are able to synthesize OT locally and exert either autocrine or paracrine effects (Gimpl and Fahrenholz, 2001). With respect to skin, it is likely that keratinocytes are candidates for local synthesis of OT. Tactile simulation to keratinocytes mediates OT release (Denda et al., 2012). We found that CQ could evoke OT but not AVP release in skin and that V1AR blockage markedly alleviated CQ-induced itch. It is suggested that endogenous skin OT mediates CQ-induced itch by acting on skin terminals of nociceptors or axonal terminals directly because V1AR is allocated at sensory terminals of DRG neurons. In conclusion, intradermally injected OT boosts CQ-induced scratching behavior *via* binding V1AR and triggering NO release (**Figure 7**). Local OT synthesis within the skin is also involved in CQ-induced scratching behavior. V1AR antagonists may provide promising therapies to treat CQ-evoked itch.

## Data Availability Statement

The datasets generated for this study are available on request to the corresponding author.

## Ethics Statement

The animal study was reviewed and approved by the committee for animal ethics and experiments at Shandong University, Jinan, China.

## Author Contributions

RL and HS performed research, analyzed the data, and helped the draft manuscript. HZ, ZZ, JL, YL, TM, and SL performed research and analyzed the data. CW and JXL were responsible for the design of the study, interpretation of data, and writing of the manuscript.

## Funding

This work was supported by grants from the National Natural Science Foundation of China (31571183, 81573904, 81401861), National Key Research and Development Program of China (2016YFC1302203), Shandong Key Research and Development Program (2017GSF218032), the Natural Science Foundation of Shandong Province (ZR2014HQ066) and Sci-tech Development Program of Department of Science & Technology of Shandong Province (2016WS0743).

## Conflict of Interest

The authors declare that the research was conducted in the absence of any commercial or financial relationships that could be construed as a potential conflict of interest.

## Abbreviation

OT, oxytocin; CNS, central nervous system; AVP, arginine vasopressin; ID, intradermal injection; CQ, chloroquine; iNOS, inducible nitric oxide synthase; DMSO, dimethyl sulfoxide; ELISA, enzyme-linked immunosorbent assay; Mrgprs, Mas-related G protein-coupled receptors; OTR, oxytocin receptor; V1AR, vasopressin-1a receptor; PLCβ, phospholipase Cβ; Ca^2+^]_i_, intracellular Ca^2+^; NO, nitric oxide; DRG, dorsal root ganglia.
