# Supplementary figures and images for: Intradermal Injection of Oxytocin Aggravates Chloroquine-Induced Itch Responses via Activating the Vasopressin-1a Receptor/Nitric Oxide Pathway in Mice
Source: Front Pharmacol. 2019 Nov 15;10:1380. doi: 10.3389/fphar.2019.01380 (PMC6881818; doi:10.3389/fphar.2019.01380)

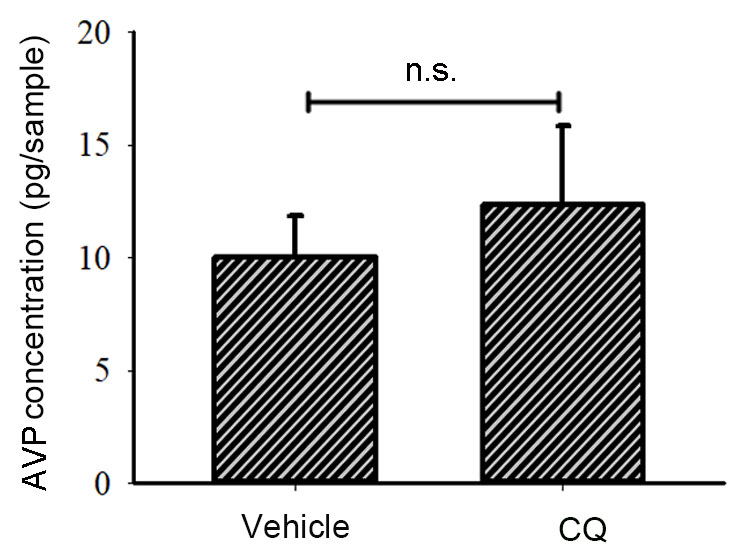

Supplement: Supplementary file 11 [file Image_1.jpeg]
